# UVC Box: An Effective Way to Quickly Decontaminate Healthcare Facilities’ Wheelchairs

**DOI:** 10.3390/life14020256

**Published:** 2024-02-16

**Authors:** Cloé Adam, Marius Colin, Romuald Stock, Laurent Weiss, Sophie C. Gangloff

**Affiliations:** 1 Université de Reims Champagne-Ardenne, UR 4691 Biomatériaux et Inflammation en Site Osseux (BIOS), UFR de Pharmacie, SFR CAP-Santé, 51 rue Cognacq Jay, 51100 Reims, Francesophie.gangloff@univ-reims.fr (S.C.G.); 2 Université de Lorraine, LCOMS, EA7306 Lorraine, France; romuald.stock@univ-lorraine.fr; 3 Université de Lorraine LEM 3, UMR CNRS 7239, 7 Rue Félix Savart, 57073 Metz, France; laurent.weiss@univ-lorraine.fr

**Keywords:** UVC, decontamination, antibacterial, healthcare-associated infections, wheelchair

## Abstract

Disinfection in the hospital environment remains challenging, especially for wide and structurally complex objects such as beds or wheelchairs. Indeed, the regular disinfection of these objects with chemicals is manually carried out by healthcare workers and is fastidious and time-consuming. Alternative antibacterial techniques were thus proposed in the past decades, including the use of naturally antimicrobial UVC. Here, the antibacterial efficiency of a large UVC box built to accommodate wheelchairs was investigated through testing bacterial burden reductions on various parts of a wheelchair, with various support types and with several treatment durations. The results demonstrate a time-dependent antibacterial effect, with a strong burden reduction at only five minutes of treatment (>3-log median reduction in *Escherichia coli* and *Staphylococcus epidermidis*). The UVC flux and residual bacterial burden both significantly varied depending on the spatial location on the wheelchair. However, the nature of the support impacted the antibacterial efficiency even more, with residual bacterial burdens being the lowest on rigid materials (steel, plastics) and being the highest on tissue. On metallic samples, the nature of the alloy and surface treatment had various impacts on the antibacterial efficiency of the UVC. This study highlights the efficiency of the tested UVC box to efficiently and quickly decontaminate complex objects such as wheelchairs, but also gives rise to the warning to focus on rigid materials and avoid porous materials in the conception of objects, so as to ensure the efficiency of UVC decontamination.

## 1. Introduction

More than ever, the spread of pathogens in human or animal communities is concerning. The COVID-19 pandemic brought the problem of microorganism’s transmission to light, especially since the rate of healthcare-associated infections (HAIs) caused by other pathogens significantly rose (eventually by 60%) during the period between March and September 2020 [1]. This sheds light on the importance of the transmission of microorganisms and how quick the spread between people can be. Since 2020, reinforced hygiene habits have been adopted by populations and numerous solutions to reduce sanitary risks in human environments have been proposed.

In healthcare facilities particularly, the protection of patients, residents, and visitors, but also healthcare workers against pathogen transmission is essential. Indeed, the prevalence of HAIs does not decrease to less than 4–5% [2]. In palliative units, this rate can dramatically rise to above 70% [3], and an important part of HAIs might originate from cross-contamination [4].

Cross-contamination between colonized and naïve persons can occur via direct contact but also through the surrounding inert environment [5]. Indeed, numerous pathogens can spread on healthcare surfaces, where they can potentially persist for months [5,6]. Thus, these contaminated surfaces represent a threat toward future users, with the contaminations being higher for frequently touched surfaces or items near the patient [5,7].

Currently, reducing the risk from environmental surfaces mainly involves chemical cleaning and manual disinfection by workers. While the antimicrobial activity of numerous chemicals agents is well known, with various efficiencies (a 5-log reduction of the microbial burden obtained within minutes to hours) [8], this type of disinfection represents a time-consuming and fastidious task. In particular, geometrically complex objects such as wheelchair, beds, and stretchers need to be frequently disinfected, which takes considerable time through manual cleaning. In addition, several studies have demonstrated that room cleaning is often insufficient, with the majority of surfaces not being properly disinfected [9,10,11,12,13,14,15].

Thus, automating the disinfection process might allow saving time and ensure a constant quality level of the process. Self-disinfecting surfaces have gained popularity over the last decades, especially copper-containing surfaces that demonstrated a microbial burden log-reduction within under 30 min to several hours in vitro [16,17] and lowered contamination levels as observed in in-use studies of copper-containing touch surfaces [17,18,19]. However, as the antimicrobial effect of copper relies on copper ions releasing from the surface through oxidation [16], the use of copper might not be adapted to the production of large objects meant to be in prolonged contact with patients’ skin.

Another promising way to perform an automated disinfection might be through ultraviolet C (UVC) treatment. For several years, UVC has drawn attention as an interesting non-chemical method to eliminate bacteria, viruses, and fungi, with promising results [20,21]. For example, a 15 s exposure reduced burdens of methicillin-resistant *Staphylococcus aureus* and of bacteriophage MS2 by more than 99% and reduced burdens of *Clostridium difficile* by 96% [20]. UVC is a non-visible electromagnetic radiation whose wavelengths range from 100 nm to 280 nm, and the 200–280 nm window is effective for microbial decontamination [22]. UVC directly interacts with DNA strands in living organisms, inducing the photodimerization of thymine, which leads to the binding of consecutive DNA bases together [23,24]. This process induces the corrupted translation and replication of the DNA. Conjointly, UVC triggers a hyperproduction of reactive oxygen species (ROS) [25]. Thus, high doses of UVC induce a large amount of mutations and an important oxidative stress that together lead to cell death (or virus inactivation) [23].

The potential applications of UVC are various, including the antimicrobial treatment of air, water, food, and inert surfaces [9,23,26,27,28]. In healthcare, small UVC boxes are already proposed for the disinfection of small items like medical ultrasound probes. While the use of UVC to disinfect larger objects is interesting, the use of UVC as a disinfection process encounters limitations that are inherent to the technology. This includes the rapid decrease of UVC energy with distance from the source (especially in water), and the inability to pass through physical barriers, which can be numerous in large and complex objects. This might make some areas unreachable for UVC, leading to incomplete disinfection, which combines with the difficulty to obtain powerful-enough UVC sources to ensure complete elimination of microorganisms on these types of wide and geometrically complex objects.

The aim of this study was to characterize the antibacterial capacities of a large UVC box (BYOLA Hosta 900^®^, Byola, Faulquemont, France) composed of powerful lamps and highly reflective inner surfaces, against *Escherichia coli*, *Staphylococcus epidermidis*, and *Bacillus subtilis* spores. The investigations examined the ability of the box to reduce the bacterial burden on various sites of a wheelchair, on various types of surfaces, and on materials of different natures.

## 2. Materials and Methods

### 2.1. Characteristics of the UVC Box

**Figure 1:**
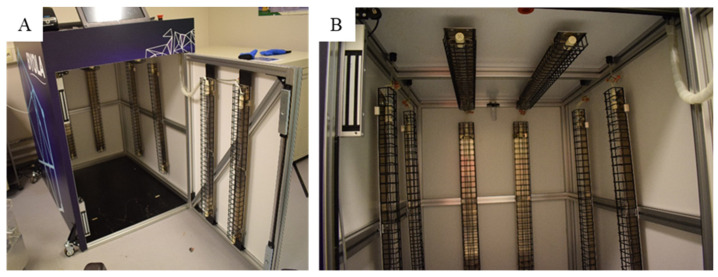
Images of the BYOLA Hosta 900^®^ UVC box. (**A**) Outside view of the box with door opened. (**B**) Inner view of the box and distribution of the UVC lamps.

The UVC box used in the tests was a BYOLA Hosta 900^®^ (BYOLA, Faulquemont, France) (Figure 1). The inner surfaces of the box measured H115×L90×D118 cm. Ten Amalgam T6 TUV XPT SE UVC lamps (Philips, Amsterdam, The Netherlands) (130 W each, peak radiation at 253.7 nm) were distributed on inner sides of the box, with two lamps per side (including door side) fixed vertically, and two lamps on the top panel, fixed horizontally. The inside panels of the box were white and non-reflecting on the four sides and top side. The floor panel was black and non-reflecting. The UVC lamps were fixed on reflecting metallic rails.

### 2.2. Wheelchair and Material Samples Preparation

**Figure 2:**
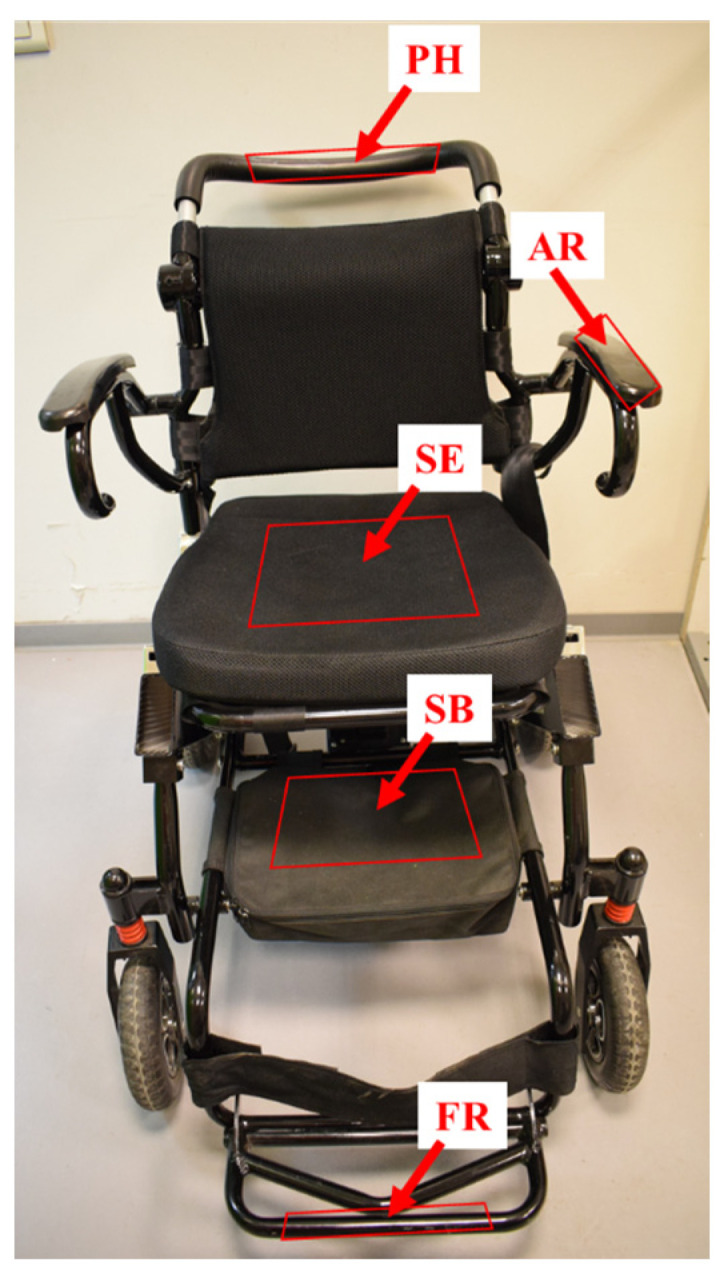
Image of the Lightchair^®^. Five positions were defined as the push handle (PH), armrest (AR), seat (SE), under-seat bag (SB), and footrest (FR).

A Lightchair^®^ (Logo Silver, Échirolles, France) wheelchair was used to perform either direct inoculation of bacteria on the wheelchair or to position material samples artificially contaminated with bacteria. Five positions were selected for bacterial tests (Figure 2). Eight types of material samples were used (Table 1).

### 2.3. Metallic Samples Preparation

Three metals used in the manufacturing of wheelchair parts were tested: 304L steel, S235 steel, and 5754 aluminum. The chemical compositions of these alloys are given in Table 2.

The samples were cylindrical discs of 5 mm thickness and 29 mm diameter. All samples were cut using a water jet from 500 × 500 × 5 mm plates in order to avoid any heating and therefore any formation of oxides.

The samples were then separated into three batches, each undergoing a different surface preparation:

- Polished samples (Pol.) to simulate a scratched condition. The specimens were polished with a polishing turret and 80# diamond abrasive paper.
- Sandblasted samples (San.) to simulate a surface condition typically found on industrial parts. Blasting was performed with a 220# corundum and 7 bar dry air pressure.
- Mirror-polished samples (Mir.) to simulate a perfect finish. The samples were manually polished to 1200 grit before being automatically polished with 9, 6, and 3 µm diamond powder solutions for 5 min at a pressure of 20 N. The final mirroring step was performed using a colloidal silica suspension (OPS) for 5 min at a pressure of 10 N.

**Figure 3:**
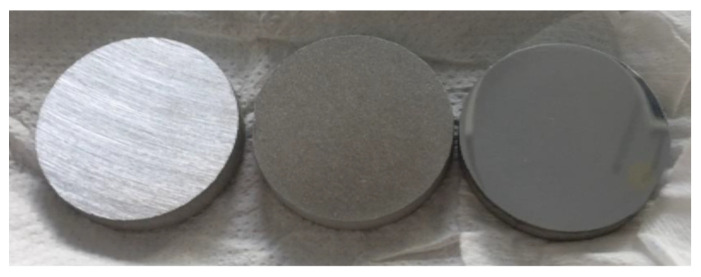
From left to right: example of S235 samples, scratched, sanded, and mirror-polished, respectively.

Figure 3 shows an overview of the processed samples. Roughness was measured using a Surftest SJ-210 roughness tester (Mitutoyo, Kawasaki, Japan) with a feed rate of 0.5 mm/s.

### 2.4. Bacterial Strains Preparation

#### 2.4.1. Vegetative Bacteria

*Escherichia coli* CIP 54.8T and *Staphylococcus epidermidis* CIP 53124 strains were maintained for long-term conservation at −80 °C and thawed just before use. Strains were seeded onto tryptic soy agar plates (TSA, Biokar Diagnostics, Allonne, France) and incubated aerobically at 37 °C for 24 h to create a reference petri dish. For each assay, two or three colonies were resuspended in 10 mL of tryptic soy broth (TSB, Sigma-Aldrich, Dutscher, Brumath, France) and incubated at 37 °C, stirring overnight. Subsequently, 3 mL of the culture was added to 50 mL of TSB, and incubated at 37 °C, stirring for four hours. After three washes with 10 mL peptone water and centrifugation (4500× *g* for 5 min), the bacterial pellet was resuspended in peptone water to reach a concentration of 10^10^ CFU/mL.

#### 2.4.2. Bacillus Subtilis Spores

A *Bacillus subtilis* ATCC 6633 spores BAS E9 suspension (Liofilchem, Roseto degli Abruzzi, Italy) was used at a final concentration of spores in the suspension of around 10^9^ CFU/mL. The initial suspension was diluted to half in distilled water and stocked at 4 °C. Before the assays, the diluted suspension was vortexed for 2 min, ultrasonicated in a bath (45 kHz) for 5 min, and the suspension was once again vortexed for 2 min. The presence of spores and absence of vegetative bacteria in the suspension were confirmed by Malachite green staining and observation by optical microscopy.

### 2.5. UVC Treatment Procedure and Evaluation of Antibacterial Activity

All types of surfaces tested were sprayed with 70% ethanol and allowed to dry for at least ten minutes before the beginning of the assay.

Three independent droplets of 10 µL of bacterial or spore suspension were deposited on the tested surface or on the material sample. The wheelchair was placed in the box and the door was sealed. The UVC lamps were then activated for 5, 15, or 30 min. The UVC power was measured using a UVC light meter (UVC-254SD, Lutron, Coopersburg, Pennsylvania, USA). After the treatment, residual bacteria in each inoculum were carefully harvested using a sterile swab moistened with 50 µL of peptone water. The swab was firmly applied to the area of the inoculum and rotated. The swab was then placed in a 50 mL sterile tube containing 7.5 mL of peptone water. Tubes were placed in an ultrasonic bath (35 Hz) for 2 min and then briefly vortexed for 20 s. Serial dilutions of each tube suspension were performed in peptone water and 100 µL of each dilution was exponentially seeded (easySpiral Pro, Interscience, Saint-Nom-la-Bretèche, France) on TSA. Plates were incubated for 24 h, either at 37 °C for the bacteria or 30 °C for the spores. Colonies were counted using the Interscience Scan 1200 to determine the number of colony-forming units (CFU) per inoculum. For the control condition, the procedure was identical, except that the UVC lamps were not activated. All biological tests were performed three times (n = 3).

### 2.6. Statistical Analysis

Conditions were compared using the non-parametric Kruskal–Wallis (KW) test for the comparison of multiple conditions and the non-parametric Mann–Whitney (MW) test (two-tailed) for the comparison of two conditions. Differences between conditions were considered significant for *p* < 0.05.

## 3. Results

### 3.1. UVC Power Displayed in the Box

**Figure 4:**
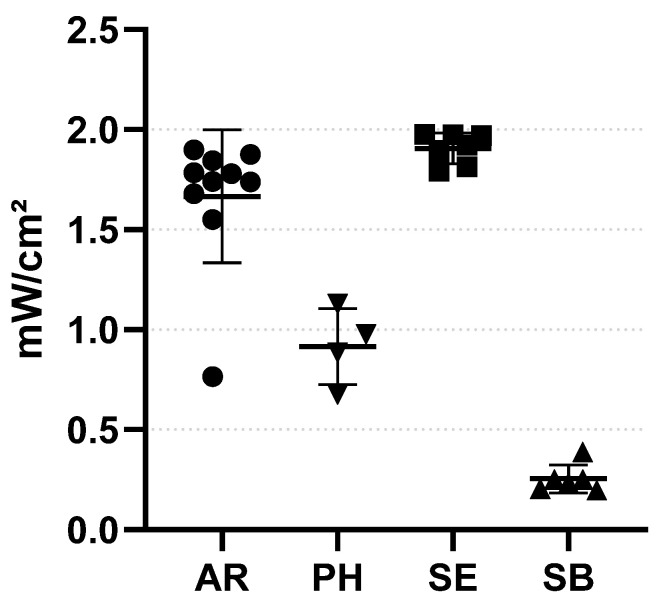
Radiant flux received at four different positions on the wheelchair placed in the Byola Hosta 900^®^ box. Light meters were positioned on the armrest (AR, dots, n = 10), push handle (PH, downward triangles, n = 4), seat (SE, squares, n = 7), or under the seat bag (SB, upward triangles, n = 6). The UVC was active for 5 min.

Important differences were observed regarding the position of the light meter on the wheelchair (Figure 4). The mean radiant flux was 1.666 mW/cm^2^ on AR, 1.906 mW/cm^2^ on SE, 0.255 mW/cm^2^ on SB, and 0.916 mW/cm^2^ on PH. The most important difference was between SB and SE, the radiant flux on SB representing only 13% of the radiant flux on SE.

### 3.2. Effects of Treatment Time and Positions on the Wheelchair

**Figure 5:**
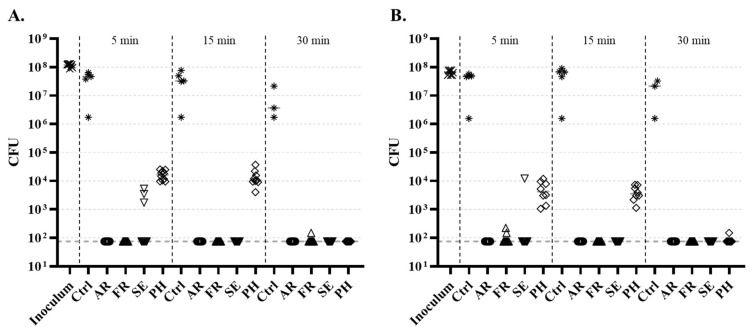
Effect of different times of UVC treatment on (**A**) *E. coli* and (**B**) *S. epidermidis* deposited on the armrest (AR, dots), footrest (FR, upward triangles), seatbelt buckle set on the seat (SE, downward triangles), and push handle (PH, diamond) of the wheelchair. Bacteria unexposed to UVC were used as control (Ctrl, stars). Burdens of initial inoculum were determined (crosses). Medians are indicated. Horizontal doted black lines indicate the detection limit (75 CFU). All conditions were significantly different from the respective unexposed control (Ctrl).

The first investigations were performed through direct inoculation of *E. coli* or *S. epidermidis* on the wheelchair and the testing of three treatment duration: 5, 15, and 30 min (Figure 5). A strong and significant antibacterial effect was observed on all surfaces and for both bacterial strains, as soon as after 5 min of treatment. However, differences were noted depending on the position on the wheelchair. Regarding *E. coli*, no surviving bacteria were detected on AR nor on FR after 5 min, but three inocula on SE and all nine inocula on PH harbored surviving bacteria. A 15 min treatment eliminated all detectable bacteria on SE but not on PH, with still around 10^4^ UFC per inocula. A 30 min treatment induced more than a 4-log reduction of the bacterial burden at all positions. The results on *S. epidermidis* were very similar, except that at 5 min a slight survival of bacteria was noted on FR.

### 3.3. Investigation of the Effect of the Surface Material and Position on the Wheelchair on the Antibacterial Activity

**Figure 6:**
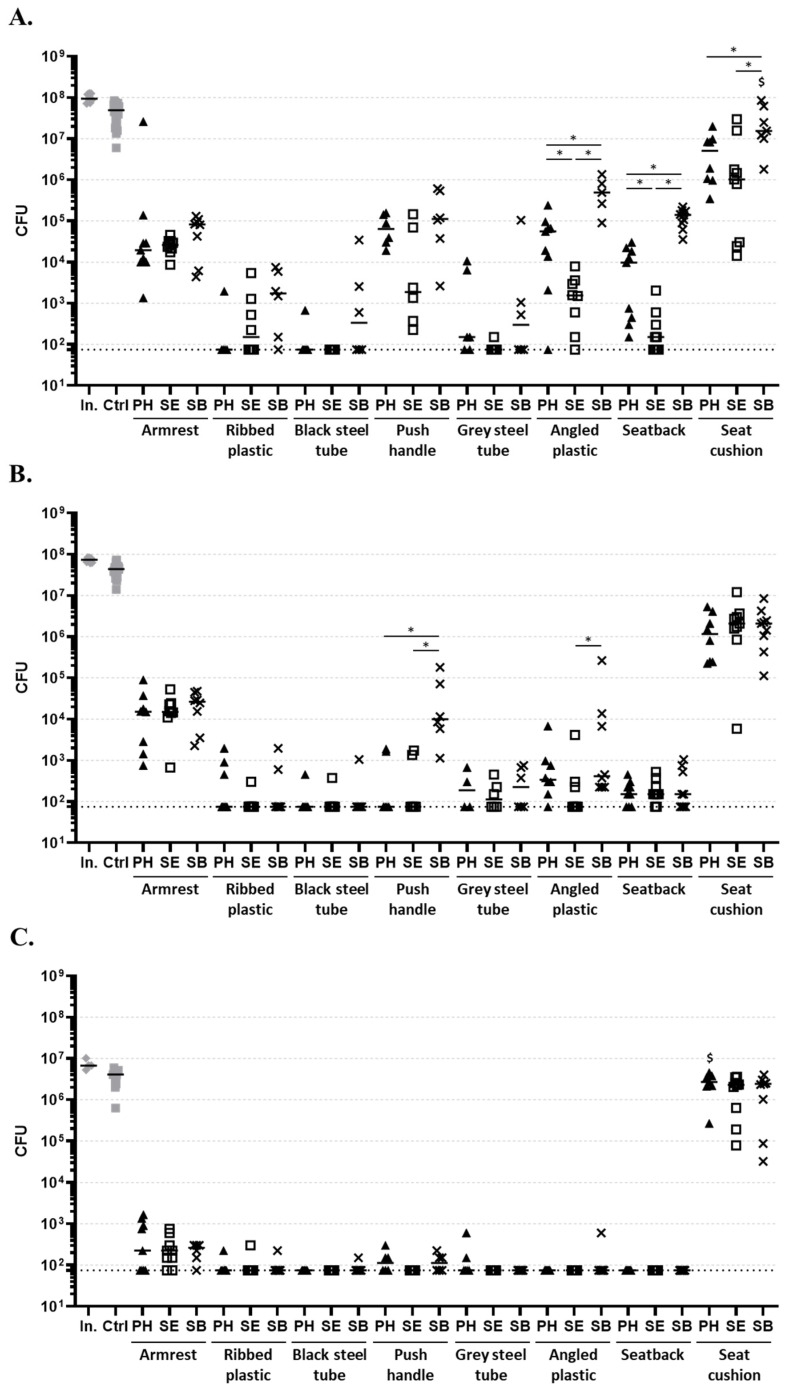
Impact of the type of material samples and their position on the reduction of bacterial burden during a 5 min UVC treatment. Samples of the eight types of materials were artificially contaminated with (**A**) *E. coli*, (**B**) *S. epidermidis*, or (**C**) spores of *B. subtilis* and placed at three different positions (PH (triangles), SE (empty squares), SB (crosses)) on the wheelchair. Burdens of initial inoculum were determined (In., grey diamonds). Bacteria unexposed to UVC were used as control (Ctrl, grey full squares). Medians are indicated. The horizontal doted black lines indicate the detection limit (75 CFU). “In.” refers to the bacterial inoculum. All conditions were significantly different from the unexposed control (Ctrl), except for conditions marked with “$”. * indicates *p* < 0.05 when comparing the same material samples at different positions (MW test).

To discriminated if the differences in bacterial survival were due to the position on the wheelchair or to the material composition of the surface, eight types of material samples cut from a second wheelchair (Table 1) were artificially contaminated with bacteria, placed at one of three positions of the wheelchair and treated with UVC for five minutes (Figure 6).

The five-minute treatment induced a statistical reduction of the bacterial burden for each strain, position, and material type (except for *E. coli* on the seat cushion at position SB and for *B. subtilis* spores on seat cushion at position PH). Overall, bacterial burdens were reduced by up to more than five logs through the treatment. Once again, the activity was stronger on *S. epidermidis* than on *E. coli* on each type of support. Surprisingly, the antibacterial effects observed on *B. subtilis* spores were higher than the effect on both *E. coli* and *S. epidermidis*, excepted for the seat cushion, on which almost no reduction was observed (0.2 log comparing medians).

While the bacterial burdens were differently impacted by the position for the push handles (*S. epidermidis*), angle plastics (*E. coli* and *S. epidermidis*), or seat back (*E. coli*), the position on the wheelchair led to undetectable differences in the antibacterial effects for most of the materials tested (e.g., no observable difference for the armrest, regardless of the strain).

Conversely, the antibacterial effect highly depended on the type of material. For example, on the seatback materials and angled plastic, residual *E. coli* burdens were significantly different between positions, the lowest burdens being observed at SE and the highest at SB.

To sum up, for each bacterial species tested (*E. coli*, *S. epidermidis*, and *B. subtilis* spores) and for each position independently, the type of material had a significant impact on the residual bacterial burden (KW test; *p* < 0.0001). The statistical one-by-one comparison of material types (Table A1) demonstrated numerous differences. In particular, the bacterial burdens from the seat cushion were significantly higher than from every other material and for each strain tested. Arm rest materials also frequently presented significant differences compared to other materials, especially black steel tubes (Table A1, Table A2 and Table A3, Appendix A).

### 3.4. Effect of Sample Surface Treatment on Antibacterial Efficacy

Metal surface texturations are known to differently influence bacterial colonization. Therefore, three treatments of three different alloys were investigated. The averaged results of the roughness analyses of the five samples are given in Table 3 below.

For steels, the results are those classically expected, i.e., surface blasting creates the highest roughness and mirror polishing the least (a few tens of nm). For aluminum, on the other hand, the Ra of polished samples is the most important. This is probably because 5754 aluminum is a softer material than steel and therefore 80# paper pulls out more chips during abrasion. Sandblasting, on the other hand, will tend to close the surface roughness and thus slightly decrease the roughness.

It should be noted that X-ray measurements did not reveal any phase transformation in the microstructure before and after surface preparation, which eliminates a possible bias during the various biological campaigns.

**Figure 7:**
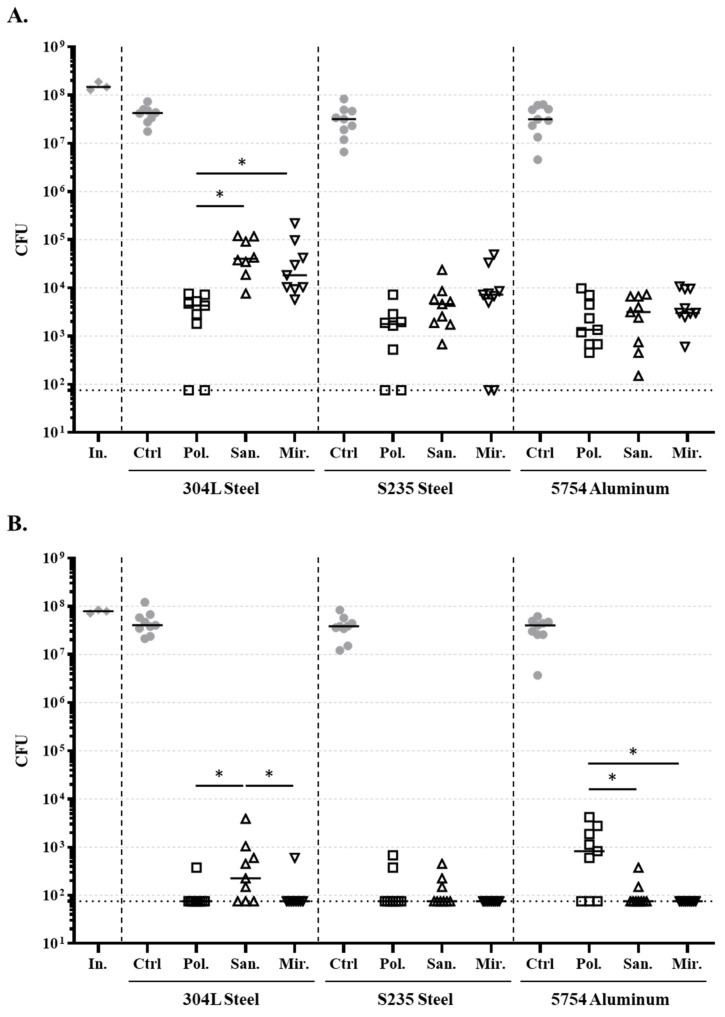
Impact of the metallic sample surface texturation on the reduction of bacterial burden during a 5 min UVC treatment. Samples of three metallic alloys (304L steel, S235 steel, and 5754 aluminum) presenting three different surface texturations (polished (Pol., empty squares), sandblasted (San., empty upward triangles), and mirror-polished (Mir., empty downward triangles)) were artificially contaminated with (**A**) *E. coli* or (**B**) *S. epidermidis* and placed on the SB position of the wheelchair. Burdens of initial inoculum were determined (In., grey diamonds). Controls (Ctrl, grey dots) were observed on metallic samples presenting one of the three texturations and unexposed to UVC treatment. Medians are indicated. The horizontal doted black lines indicate the detection limit (75 CFU). “In.” refers to the bacterial inoculum. All conditions were significantly different from the respective unexposed control. * indicates *p* < 0.05.

UVC antibacterial effects were investigated on *E. coli* and *S. epidermidis* inoculated on these nine surface types (Figure 7). The bacterial burden reduction ranged from two to five logs for *E. coli* and from three to five logs for *S. epidermidis* with, overall, less residual bacteria for *S. epidermidis* after the UVC treatment.

Considering *E. coli*, the polished texturation demonstrated the lowest bacterial burden, while the sandblasted and mirror texturations were similar. Notable differences were observed between alloys, with 304L steel demonstrating higher levels of residual bacteria than the two other materials. For *S. epidermidis*, trends were different from *E. coli*. This was especially noticeable for 5754 aluminum, for which the highest residual burdens were observed for the polished texturation.

## 4. Discussion

Since the last decades, UVC has risen as a promising and effective tool to decontaminate air, water, food, materials, and fomites [23]. While the technology is attractive thanks to its high antimicrobial efficiency/treatment time ratio, UV rays are quickly blocked by physical barriers, and proofs of UVC disinfection efficiency on complex items are still needed. The present study aimed to examine the antibacterial efficiency of a large UVC box dedicated to wheelchair disinfection in care centers.

The evaluation of UVC doses in the box through treatment cycles clearly demonstrated that UVC reached all four positions tested (AR, PH, SE, and SB). However, the radiant flux highly differed from one position to another, almost reaching a ten-time difference between the lowest (SB) and the highest (SE) positions. These differences might be dependent of two factors. First, the position, orientation, and reflectance of the surface presenting the inoculum with regard to the UVC lamps can strongly influence the irradiance received by this surface [29]. Second, the wheelchair structure can act as a barrier toward UVC rays, thus reducing the overall flux in inward zones such as SB. The first bacteriological tests performed directly on wheelchair surfaces demonstrated that the antibacterial effect was, indeed, stronger at positions exposed to stronger flux, with less residual bacteria at SE than at PH. Also, a time-dependent effect was observed, and while most bacteria were eliminated during the first five minutes of treatment (at least a 1.5-log reduction), a 4- to 5-log eradication in extremely concentrated bacterial burdens might require up to 30 min.

These tests indicated that some factors importantly impact the efficiency of the disinfection process, but it was unclear if the position in the box, the material nature, or both were the cause. The cross-tests on every material at three different positions clearly demonstrated the influence of the material nature on the antibacterial effect. This was especially evident with *E. coli* at the SE position: while most of the material displayed less than 10^4^ CFU, all values from the seat cushion were higher than 10^4^ CFU and even reached 3.10^7^ CFU. Moreover, the seat cushion was systematically the surface presenting the most residual bacteria, regardless of the bacterial strain or the position in the box. These results are not surprising, as the seat cushion is made up of porous materials, allowing bacteria to infiltrate inside and blocking UVC rays. In a less significant way, the armrest also displayed specific responses. While the material was non-porous, the overall bacterial burdens on the armrest were higher than on most other non-porous samples, no complete bacterial elimination was observed for *E. coli* and *S. epidermidis* tests, and almost no differences were observable between positions. These intriguing results support the huge impact of the material nature on the UVC decontamination efficacy and suggest that the conception of objects meant to be frequently decontaminated by UVC might avoid specific materials such as leather/false leather and, above all, porous materials.

While results differed between *E. coli* and *S. epidermidis*, surface texturation also seems to have an impact on the antibacterial effect. However, no clear overall effect was observed. Indeed, the polished samples demonstrated the lowest residual charge, but only for *E. coli*, while the mirrored samples demonstrated the best results for *S. epidermidis*. In the present study, texturation was only performed on metallic samples, but further investigation on other materials is needed to understand if texturation may prevail on the material composition and in which cases. The surface reflectiveness has also been pointed out to play an important role in treatment efficiency [30].

Among all bacteriological tests, some were surprising. Indeed, for the same conditions, *E. coli* appeared more resistant to UVC than *S. epidermidis*, while Gram-negative bacteria are known to be more sensitive than Gram-positive bacteria [31]. These differences may be explained through using different strains and even species (*Staphylococcus aureus* vs. *S. epidermidis*) between studies. Also, the UVC wavelengths are often different between studies: Kim et al. [31] tested wavelengths ranging from 266 to 279 nm, while lamps essentially emitting at 253.7 nm were used in the present study. It is possible to hypothesize that some bacteria species or strains may be more sensitive to some wavelengths than others [32]. Very interestingly, *B. subtilis* spores did not show a specific survival compared to vegetative strains, except for tests on the seat cushion. Nevertheless, the UVC doses recorded in the box ranged from 59.7 to 592.5 mJ/cm^2^, which is 10 to 100 times the amount of energy needed to reduce the burden of *B. subtilis* ATCC6633 by one log, according to Nicholson and Galeano (2003) [33]. Thus, the important reduction (and even total elimination in most of the cases) of spores seems consistent. Other studies have already demonstrated various responses from one bacterial strain to another with, sometimes, higher resistances in vegetative bacteria [34] than in spores [35], confirming that the type of UVC lamps, the conception of the box, and the nature of materials to be decontaminated are, among others, crucial parameters influencing the treatment efficiency. Thus, each new box prototype should be tested for its antimicrobial abilities, under multiple experimental conditions.

To summarize, the results obtained in this study are promising on non-porous materials, with reductions that range from 0.25 to more than 5 logs after five minutes of treatment. While it is hard to compare with other disinfection methods due to the numerous differences in study protocols, these reduction levels seem consistent with the ones obtained with various chemical disinfectants that require several minutes to several hours to eliminate three to five logs of the microbial burden [8]. A Canadian study demonstrated that manual disinfection appeared fastidious and unclear, and that wheelchairs were not disinfected between two patients in around 50% of cases [36]. Thus, UVC disinfection appears interesting for the disinfection of complex models like wheelchairs, so as to save both chemical costs and worker time.

One limitation that can be addressed regarding the box tested here is the use of mercury lamps as the UVC source. Indeed, over the past few years, UVC LEDs have gained popularity because of their advantages including robustness, a longer lifecycle, and the absence of hazardous materials. However, UVC mercury lamps still have a better wall-plug efficiency [23], which renders them a better choice for decontamination in large containers, whereas UVC LEDs require much more electrical energy [23,37,38]. Nevertheless, like any other method of disinfections, as dirt residues might act as a physical barrier that protect microorganisms, another limitation to the use of UVC lamps to decontaminate surfaces remains the necessity to clean the surface prior to the UVC treatment.

Another obstacle to UVC use in disinfection is the long-term modifications of the UVC-treated surfaces. Indeed, repeated radiation treatments can have an influence on surfaces colors and microstructures through years of disinfection cycles [39]. While these effects do not compromise wheelchair integrity nor UVC treatment effectiveness, it would be interesting if further studies focused on surfaces that underwent numerous disinfection cycles. This will ensure the maintaining of the optimal functioning of the UVC on “long-term use” wheelchairs that underwent repeated treatments.

Furthermore, the inside of the box model was made of non-reflecting surfaces. The use of reflecting surfaces might help avoid UVC loss on absorbing surfaces and increase the UVC quantities actually reaching the wheelchair parts. Thus, this would probably increase the antimicrobial efficiency of the box and reduce the time and energy displayed to reach a satisfying reduction of microbial burdens.

The next step of the investigations will be to validate the efficiency of the UVC box through in situ study through the environmental sampling and analyses of microbial flora colonizing wheelchairs in hospital care units. Further, as the patient benefit represents the fundamental goal, studies must focus on epidemiological investigation in UVC box-equipped versus non-equipped units, to prove the concrete advantage for patients’ health. While this type of study exists [40], evidence needs to accumulate to guarantee UVC’s assets in healthcare.

## 5. Conclusions

In conclusion, the BYOLA Hosta 900^®^ UVC box displayed interesting antibacterial action, reducing the bacterial burden of both vegetative bacteria (both Gram-positive and Gram-negative) and spores. While a duration as short as five minutes might be enough for a routine decontamination of wheelchairs, the efficiency of the treatment highly relies on the material nature. UVC treatment is ineffective to confidently decontaminate porous materials; thus, only non-porous materials should be used for the conception of wheelchairs adapted to this type of box. As non-porous materials also displayed various responses regarding the efficiency of decontamination, further studies are needed to precisely characterize the interactions between UVC, bacteria, and different supports of different natures. Overall, large UVC boxes might represent an effective solution to quickly reduce the microbial burden and disinfect wheelchairs or other complex and large pieces of equipment, allowing both to standardize the procedure and to save workers’ time.

## Figures and Tables

**Table 1:** Material samples from wheelchair.

Type of Samples	Materials	Porosity
Armrest	Artificial leather	Non-porous
Push handle	Rubber	Non-porous
Black steel tube	Painted steel	Non-porous
Grey steel tube	Painted steel	Non-porous
Ribbed plastic	Plastic	Non-porous
Angled plastic	Plastic	Non-porous
Seatback	Artificial leather	Non-porous
Seat cushion	Tissue and foam	Porous

**Table 2:** Chemical compositions (wt%) of 5754 aluminum, S235 steel, and 304L steel.

**5754 Aluminum**	Al	Mg	Mn	Fe	Si	Cr	Zn	Ti	Cu
	bal.	2.6–3.6	<0.5	<0.4	<0.4	<0.3	<0.2	<0.15	<0.1
**S235 steel**	Fe	C	Mn	Cu	P	S	N		
	bal.	<0.2	<1.4	<0.55	<0.04	<0.04	<0.012		
**304L steel**	Fe	C	Cr	Ni	Mn	Si	N	P	S
	bal.	<0.03	17.5–19.5	8–10.5	<2	<1	<0.11	<0.045	<0.015

**Table 3:** Roughness Ra of the surface-prepared samples.

	304L Steel			S235 Steel			5754 Aluminum		
	Pol.	San.	Mir.	Pol.	San.	Mir.	Pol.	San.	Mir.
**Ra (µm)**	0.584	2.061	0.029	0.832	1.305	0.01	1.651	0.887	0.032
**Standard deviation**	0.059	0.145	0.027	0.052	0.092	0.002	0.164	0.206	0.011

## Data Availability

The data presented in this study are available on request from the corresponding author (marius.colin@univ-rems.fr).

## Appendix A

**Table A1:** Statistical comparison of residual *E. coli* burdens on materials. *p* values of Mann–Whitney tests. Numbers are noted in bold when *p* < 0.05.

**PH position:**								
	Arm rest	Ribbed plastic	Black steel tube	Push handle	Grey steel tube	Angled plastic	Seatback	Seat cushion
Arm rest								
Ribbed plastic	**0.002**							
Black steel tube	**0.0004**	0.7273						
Push handle	0.1077	**0.0043**	**0.0022**					
Grey steel tube	**0.0046**	0.21	0.1515	**0.0022**				
Angled plastic	0.7125	**0.0065**	**0.0028**	0.405	**0.0162**			
Seatback	0.0892	**0.0105**	**0.0022**	**0.0016**	**0.047**	0.0625		
Seat cushion	**0.0054**	**0.0016**	**0.0007**	**0.0007**	**0.0007**	**<0.0001**	**<0.0001**	
**SE position:**								
	Arm rest	Ribbed plastic	Black steel tube	Push handle	Grey steel tube	Angled plastic	Seatback	Seat cushion
Arm rest								
Ribbed plastic	**<0.0001**							
Black steel tube	**0.0002**	0.0769						
Push handle	0.3277	**0.0443**	**0.0006**					
Grey steel tube	**0.0004**	0.1329	0.4615	**0.0022**				
Angled plastic	**<0.0001**	0.0993	**0.0014**	0.662	**0.007**			
Seatback	**<0.0001**	0.8566	**0.0337**	**0.01**	0.168	**0.0355**		
Seat cushion	**0.0315**	**<0.0001**	**0.0002**	**0.012**	**0.0004**	**<0.0001**	**<0.0001**	
**SB position:**								
	Arm rest	Ribbed plastic	Black steel tube	Push handle	Grey steel tube	Angled plastic	Seatback	Seat cushion
Arm rest								
Ribbed plastic	**0.0047**							
Black steel tube	**0.0027**	0.4978						
Push handle	0.4136	**0.0087**	**0.0043**					
Grey steel tube	**0.0193**	0.3268	>0.9999	**0.0087**				
Angled plastic	**0.0109**	**0.0043**	**0.0043**	0.2468	**0.0087**			
Seatback	0.0592	**0.0004**	**0.0004**	0.9546	**0.0028**	**0.042**		
Seat cushion	**0.0003**	**0.0012**	**0.0012**	**0.0012**	**0.0012**	**0.0025**	**0.0002**	

**Table A2:** Statistical comparison of residual *S. epidermidis* burdens on materials. *p* values of Mann–Whitney tests. Numbers are noted in bold when *p* < 0.05.

**PH position:**								
	Arm rest	Ribbed plastic	Black steel tube	Push handle	Grey steel tube	Angled plastic	Seatback	Seat cushion
Arm rest								
Ribbed plastic	**0.0011**							
Black steel tube	**0.0007**	0.4126						
Push handle	**0.007**	>0.9999	0.4545					
Grey steel tube	**0.004**	>0.9999	0.4	>0.9999				
Angled plastic	**0.0022**	0.2351	**0.023**	0.315	0.2747			
Seatback	**<0.0001**	0.8612	0.1944	0.7385	0.8601	0.0603		
Seat cushion	**0.0002**	**0.0002**	**0.0007**	**0.0007**	**0.004**	**0.0002**	**<0.0001**	
**SE position:**								
	Arm rest	Ribbed plastic	Black steel tube	Push handle	Grey steel tube	Angled plastic	Seatback	Seat cushion
Arm rest								
Ribbed plastic	**0.0002**							
Black steel tube	**0.0003**	0.7333						
Push handle	**0.0027**	0.3187	0.3147					
Grey steel tube	**0.0007**	0.248	0.2902	>0.9999				
Angled plastic	**0.0003**	0.4462	0.4974	>0.9999	0.9394			
Seatback	**<0.0001**	**0.0211**	**0.0437**	0.5756	0.5439	0.4103		
Seat cushion	**0.0037**	**<0.0001**	**0.0002**	**0.0004**	**0.0004**	**<0.0001**	**<0.0001**	
**SB position:**								
	Arm rest	Ribbed plastic	Black steel tube	Push handle	Grey steel tube	Angled plastic	Seatback	Seat cushion
Arm rest								
Ribbed plastic	**<0.0001**							
Black steel tube	**0.0007**	0.8418						
Push handle	0,8518	**0.0004**	**0.0022**					
Grey steel tube	**0.0007**	0.4821	0.5455	**0.0022**				
Angled plastic	0.0373	**0.0081**	0.0123	0.1395	0.1868			
Seatback	**0.0007**	0.3267	0.2963	**0.0004**	0.9888	**0.0412**		
Seat cushion	**<0.0001**	**<0.0001**	**0.0004**	**0.0008**	**0.0004**	**0.0002**	**<0.0001**	

**Table A3:** Statistical comparison of residual *B. subtilis* spores burdens on materials. *p* values of Mann–Whitney tests. Numbers are noted in bold when *p* < 0.05.

**PH position:**								
	Arm rest	Ribbed plastic	Black steel tube	Push handle	Grey steel tube	Angled plastic	Seatback	Seat cushion
Arm rest								
Ribbed plastic	**0.0498**							
Black steel tube	0.0709	>0.9999						
Push handle	0.351	0.2022	0.1818					
Grey steel tube	0.2308	0.4264	0.4545	0.9242				
Angled plastic	**0.0294**	>0.9999	>0.9999	**0.044**	0.1429			
Seatback	**0.0294**	>0.9999	>0.9999	**0.044**	0.1429	>0.9999		
Seat cushion	**0.0002**	**<0.0001**	**0.0012**	**0.0012**	**0.0012**	**<0.0001**	**<0.0001**	
**SE position:**								
	Arm rest	Ribbed plastic	Black steel tube	Push handle	Grey steel tube	Angled plastic	Seatback	Seat cushion
Arm rest								
Ribbed plastic	**0.0115**							
Black steel tube	**0.0086**	>0.9999						
Push handle	**0.0086**	>0.9999	>0.9999					
Grey steel tube	**0.0086**	>0.9999	>0.9999	>0.9999				
Angled plastic	**0.0023**	>0.9999	>0.9999	>0.9999	>0.9999			
Seatback	**0.0023**	>0.9999	>0.9999	>0.9999	>0.9999	>0.9999		
Seat cushion	**<0.0001**	**<0.0001**	**0.0004**	**0.0004**	**0.0004**	**<0.0001**	**<0.0001**	
**SB position:**								
	Arm rest	Ribbed plastic	Black steel tube	Push handle	Grey steel tube	Angled plastic	Seatback	Seat cushion
Arm rest								
Ribbed plastic	**0.0056**							
Black steel tube	**0.0281**	>0.9999						
Push handle	0.1017	0.2352	0.4242					
Grey steel tube	**0.0256**	>0.9999	>0.9999	0.1818				
Angled plastic	**0.0152**	0.7353	>0.9999	0.2448	>0.9999			
Seatback	**0.002**	>0.9999	0.4	**0.044**	>0.9999	0.4706		
Seat cushion	**0.0004**	**<0.0001**	**0.0004**	**0.0004**	**0.0004**	**<0.0001**	**<0.0001**	
